# Application of convolutional neural networks for surface discontinuities detection in shielded metal arc welding process

**DOI:** 10.3389/frobt.2025.1632417

**Published:** 2025-11-27

**Authors:** Elisa Elizabeth Mendieta, Hector Quintero, Cesar Pinzon-Acosta

**Affiliations:** 1 Facultad de Ingenieria Mecanica, Universidad Tecnologica de Panama, Panama, Panama; 2 Centro de Estudios Multidisciplinarios en Ciencias, Ingenieria y Tecnologia (CEMCIT AIP), Universidad Tecnologica de Panama, Panama, Panama

**Keywords:** shielded metal arc welding (SMAW), weld quality assurance, weld surface discontinuities, convolutional neural networks, computer vision

## Abstract

Detecting surface discontinuities in welds is essential to ensure the structural integrity of welded elements. This study addresses the limitations of manual visual inspection in shielded metal arc welding by applying convolutional neural networks for automated discontinuities detection. A specific image dataset of discontinuities on Shielded Metal Arc Welding weld seams was developed through controlled experiments with various electrode types and welder experience levels, resulting in 3,000 images. The YOLOv7 architecture was trained and evaluated on this dataset, achieving a precision of 97% and mAP@0.5 of 94%. Results showed that increasing the dataset size and training periods significantly improved detection performance, with optimal accuracy observed around 250–300 epochs. The model demonstrated robustness to moderate variations in image aspect ratio and generalization capabilities to an external dataset. This paper presents an approach for detecting SMAW weld surface discontinuities, offering a reliable and efficient alternative to manual inspection and contributing to the advancement of intelligent welding quality control systems.

## 1 Introduction

Welding is an essential manufacturing process employed for the joining, repairing, and reinforcing of metal components. It comprises more than ninety processes employed in various industries (American Welding Society, 2008). The welding process selection depends on the material, joint design, required strength, available equipment, environmental conditions, etc. Among all the welding processes, Shielded Metal Arc Welding (SMAW) remains one of the most widely adopted due to its versatility, simplicity, and ease of implementation in small-scale applications (American Welding Society, 2001). Regardless of the welding process, welding discontinuities are inevitable, whether they are done manually or automatically. To ensure the quality of weldments, an efficient and accurate weld inspection plan is required (Xu and Li, 2024).

Weld quality assurance is fundamental during fabrication as it guarantees the weldment complies with the design requirements. In the SMAW process, surface discontinuities such as cracks, porosity, undercut, slag inclusion, and incomplete fusion can result at the end of the process. These discontinuities can compromise the behavior of the welded joints, leading to failures or unsafe operations. To mitigate these risks, traditional quality assurance practices include both destructive (DE) and non-destructive examinations (NDE). Unlike destructive examination, NDE methods allow the weldment inspection without damaging the component, making them ideal for in-service inspection and production control (Deepak et al., 2021). Among NDE techniques, methods such as radiographic testing (RT), ultrasonic testing (UT), magnetic particle testing (MT), dye penetrant testing (PT), and visual examination (VE) are widely used during and after fabrication to detect internal and superficial discontinuities in the weldments. Although considered the most basic inspection form, VE remains an important NDE due to its simplicity and cost effectiveness (American Welding Society, 2001; American Welding Society, 2011). However, the examination depends on human judgment that introduces variability and subjectivity, potentially compromising the reliability and accuracy of defect detection on weldments (Yang et al., 2022). In this regard, the search for automated tools for the visual inspection method has gained popularity in the manufacturing industry as they could provide an objective and repeatable assessment of weld surfaces.

In this context, advances in machine vision technology have shown great potential for quality control of surface discontinuities. Over the years, various approaches have been developed to address this task, ranging from classical image processing techniques such as edge detection, thresholding, and morphological operations to more advanced machine-based and deep learning-based models (Bhatt et al., 2021). Traditional image processing methods are often limited by sensitivity to noise, lighting conditions, and surface variability (Tsai and Huang, 2003; Jie et al., 2009; Park et al., 2009; Borghese and Fomasi, 2015). Learning-based approaches, including support vector machines (SVM) and random forests, have improved classification performance but still rely on handcrafted features and preprocessing steps (Chittilappilly and Subramaniam, 2017; Mustaqeem and Saqib, 2021; Le, 2022). In contrast, deep learning models, particularly convolutional neural networks (CNN), have demonstrated superior performance by automatically learning hierarchical representations from raw image data (Yang D. et al., 2021; Ding et al., 2022; Taheri et al., 2022; Wang et al., 2024).

Among deep learning methods, the You Only Look Once (YOLO) family of models has gained prominence due to its real-time object detection capability, high accuracy, and efficiency (Redmon et al., 2015). Successive versions of YOLO, from YOLOv1 to YOLOv8, have been applied to detect several industrial defects (Hussain, 2023). In this regard, it has been successfully implemented to detect welding discontinuities such as crack, pores, and slag inclusion in processes like Gas Metal Arc Welding (GMAW), Gas Tungsten Arc Welding (GTAW), and Laser Beam Welding (LBW) (Deng et al., 2021; Wang et al., 2024; Xu and Li, 2024). However, each welding process exhibits unique thermal profiles, surface textures, and discontinuity types, which limit the transferability of models trained on one process to others. In this regard, SMAW processes exhibit process-specific visual and statistical properties that differ markedly from wire-fed, gas-shielded processes (Lampman, 1997; Bohnart, 2017). In SMAW, flux-covered electrodes (e.g., E6010/E7018) produce variable slag formation and post-weld residues; the heat-affected zone (HAZ) heavily depends on manual operator control that introduces sample-to-sample variations in arc length, travel speed, and weave pattern, resulting in a surface that exhibits uneven bead geometry, spatter, and oxidation that change reflectance and surface texture. Recent surface vision defect detectors remain centered on gas-shielded welds (Li et al., 2023; Xu and Li, 2024; Diaz-Cano et al., 2025), leaving SMAW unaddressed and establishing a domain gap.

In gas-shielded processes, surfaces are cleaner and heat input is more stable; the HAZ is typically narrower and more consistent across passes, and its dominant discontinuities differ (e.g., cold lap/lack of fusion in short-circuit GMAW; tungsten inclusion in GTAW). LBW features extremely high power density, a very narrow HAZ, and keyhole dynamics that promote porosity and solidification cracking, with fine-scale visual signatures. These process-specific differences produce domain shifts (occlusion, spatter noise, changes in bead-geometry, and distinct color/reflectance in the HAZ) that limit the direct transfer of models trained on GMAW/GTAW/LBW datasets (Bohnart, 2017). This motivates a SMAW-specific dataset and model that accounts for slag dynamics, manual variability, and SMAW-typical discontinuities.

This study aims to develop and evaluate a YOLOv7-based detector trained on a custom SMAW surface-image dataset annotated for eight discontinuity classes (slag inclusion, porosity, undercut, overlap, crater, arc strike, underfill and spatter) with ≥ 90% precision and mAP@0.5, and to assess its performance and robustness under realistic industrial variability including operator skill, electrode type, illumination, and image aspect ratio. The analysis further examines the model’s generalization capability when applied to external SMAW images.

## 2 Materials and methods

To enable the development of an automated system for surface discontinuities in the SMAW process, it is necessary to construct a dedicated image dataset representative of the specific visual characteristics and variability associated with this process. Unlike other welding methods, SMAW weld exhibits a broad range of surface appearances due to electrode type, operator skill, and process parameters. As publicly available datasets predominantly focus on GMAW, GTAW, or LBW, this study undertook a controlled experimental campaign to generate a high-quality, diverse dataset of SMAW weld beads with intentional surface discontinuities.

### 2.1 Materials

The base material used for welding was A36 black steel, chosen for its common use in structural applications and compatibility with the selected electrodes. Steel plates of 150 mm × 50 mm x 6 mm were prepared for the welding tasks. Each plate provided a sufficient area for a single bead per electrode, ensuring clear visibility of surface characteristics and discontinuity formation.

Before welding, the plate surfaces were cleaned using an angle grinder to eliminate any contaminants that could compromise weld quality. A total of 150 steel plates were prepared for this study, with each plate receiving two weld beads, resulting in 300 individual weld beads forming the complete set of samples used for subsequent imaging, labeling, and analysis in the experimental process.

### 2.2 Welding equipment and consumables

All welding operations were performed using a Transpocket 180 Fronius welding machine. Four commonly used electrode types, E6010, E6011, E6013, and E6018, with a 3.2 mm diameter, were selected for the experiments. These electrodes differ in arc characteristics, penetration, and slag properties, thereby introducing natural variability in weld bead appearance and potential discontinuity types.

### 2.3 Welder participation

To ensure a broad representation of weld quality and surface conditions, operators with varying levels of expertise carried out the welding operations. The group included engineering students, general welders with practical field experience, and certified professional welders. This stratified approach was chosen to promote variability in weld outcomes and maximize the likelihood of capturing a diverse range of surface discontinuities across electrode types.

### 2.4 Image acquisition setup

**FIGURE 1:**
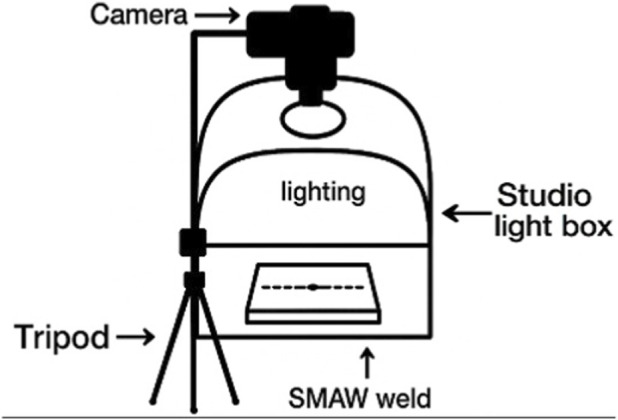
Image acquisition setup.

The visual documentation of each weld bead was carried out using a Canon EOS Rebel T8I (24.1 MP) and a Sigma 105 mm macro lens on a tripod. The camera operated in manual exposure at f/4, 1/400 s, and ISO 160, with no flash and fixed with white balance. Illumination was provided by a Godox Triple-light LED Mini Photography Studio (LST40) driven by a Godox LSC3 three-channel controller, with all three channels set to 100% output; camera and lighting geometry were held constant across sessions, while illuminance at the weld surface (lux) was not instrumented. Figure 1 and Table 1 summarize the light-box configuration and geometric setup used throughout the experiments.

**TABLE 1:** Camera and lighting parameters.

Component	Parameter	Value
Camera	Exposure mode	f/4, 1/400 s, ISO 160; WB fixed; no flash
Lighting	Model/controller	LST40 lightbox/Godox LSC3
	Output	100% on all three channels
Geometry	Mount	Tripod; fixed camera – lightbox geometry

### 2.5 Welding protocol

The welders were provided with a welding procedure specification (WPS) with the welding process characteristics (base material, filler material, joint design, welding position, welding parameters, and cleaning procedures). This procedure enabled a controlled weld generation with varied features under repeatable conditions, forming the basis for a comprehensive SMAW weld surface image dataset.

SMAW beads were produced using the Fronius Transpocket 180 power source’s preset programs, selected by coating family: CEL for cellulosic electrodes (E6010, E6011) and STICK for low-hydrogen/rutile electrodes (E7018, E6013). All deposits were bead-on-pate in the flat (1G) position with a single pass per electrode. The current window applied was E6013: 80–130 A, E7018: 90–130 A, E6010: 70–100 A, and E6011: 60–100 A; the power source automatically regulated arc voltage according to the selected program (no manual voltage setpoint). Travel speed was operator-controlled and not instrumented. Because our focus is on surface discontinuities, no joint preparation was used (bead-on-plate only); thus, lack of fusion effects tied to joint geometry are not confounders. Table 2 summarizes the SMAW setup by electrode.

**TABLE 2:** SMAW setup by electrode.

Electrode	Program	Geometry/Position/Passes	Current window (A)	Arc-voltage control	Electrode diameter (mm)
E6010	CEL	Bead-on-plate/1G/1 pass	70–100	Program controlled (voltage cannot be set manually; arc dynamics and termination voltage are adjustable through the setup menu)	3.2
E6011	CEL		60–100		3.2
E6013	STICK		80–130		3.2
E7018	STICK		90–130		3.2

### 2.6 Post welding cleaning

After welding, the plates were moved to a cleaning station to remove the slag that may have remained on the surface. For the cleaning process, the plates were secured using C-clamps, and with the assistance of an angle grinder, they were cleaned to achieve a smooth and uniform finish.

### 2.7 Image labeling process

A manual image labeling process was conducted following data acquisition to enable the supervised training of a deep learning model for weld surface discontinuity classification. For this task, eight types of SMAW discontinuities were selected: crater, slag inclusion, porosity, incomplete fusion, arc strike, underfill spatter, and undercut. The selection of these classes was based on their documented occurrence in SMAW processes and their industrial relevance according to established welding literature. The occurrence of discontinuities is described in Chapter 13, Table 13.1 of the AWS Welding Handbook (American Welding Society, 2001). Definitions and visual identification criteria were aligned with the AWS Welding Inspection Handbook (American Welding Society, 2015) and the AWS A3.0 Standard Welding Terms and Definitions (American Welding Society, 2020b).

From an industrial perspective, the acceptance criteria for many of these discontinuities are specified in Clause 8 of the AWS D1.1 Structural Welding Code–Steel (American Welding Society, 2020a), particularly Section 8.9 on visual inspection and Table 8.1, which defines dimensional and qualitative acceptance limits. In the present work, the model aims to identify the presence and type of discontinuities as an automated visual inspection tool. The decision on whether a discontinuity constitutes a defect, according to the acceptance criteria in Table 8.1 or other standards, is outside the current scope of this study.

In addition to discontinuity classification, the model was also designed to identify the weld bead as a distinct class, allowing for better contextual understanding and spatial referencing during detection. Because the proposed system is based on visual inspection, only surface-visible discontinuities were included in the data set. Subsurface or internal conditions, such as incomplete joint penetration, cannot be reliably detected through this method and were therefore excluded.

During labeling, the main challenges involved complex surface textures, overlapping or closely spaced discontinuities, and minor variations in lighting or weld orientation that could affect defect interpretation. To ensure inter-annotator consistency, a discontinuity labeling protocol was developed based on the above AWS references (American Welding Society, 2001; American Welding Society, 2011; American Welding Society, 2015; American Welding Society, 2020b; American Welding Society, 2020a). The protocol provided class definitions, visual examples, and decision rules for borderline cases, enabling annotators to follow a standardized procedure. Importantly, since the research team had access to the actual weld seams, annotators were able to visually verify the discontinuities directly on the physical specimens whenever uncertainty arose during image labeling. Prior to complete annotation, a calibration stage was conducted where annotators jointly reviewed a subset of images, compared observations with the real welds, discussed discrepancies, and refined the protocol until consensus was reached.

**FIGURE 2:**
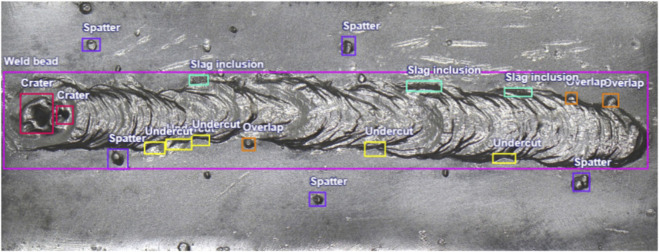
Annotated example of a SMAW weld bead image showing labeled discontinuities.

Annotation then proceeded in pairs (two annotators per pair). Within each pair, the annotator generated draft labels and then resolved discrepancies by discussion to produce pairwise consensus labels, which constitute the released dataset. Labels were created manually using open-source annotation software, following the discontinuity labeling protocol. Because labels were produced by pairwise consensus and pre-consensus disagreements were not logged, we do not report formal inter-annotator agreement for this release; future updates may include a stratified IAA on an independently re-annotated subset. We did not log pre-consensus disagreements or compute formal inter-annotator agreement metrics for this release. An example of a labeled weld bead image with annotated discontinuities is shown in Figure 2.

### 2.8 Data augmentation

To enhance the diversity of the training dataset, data augmentation techniques were applied to the original manually labeled images. Three augmentation methods were employed: 90-degree rotation, horizontal mirroring, and Gaussian filtering. Each transformation was performed systematically, and the corresponding annotation files were adjusted to preserve the discontinuity location and class labeling accuracy. Special attention was given to the spatial orientation transformation, ensuring the bounding boxes were correctly recalculated for each augmented image. The augmentation process expanded the dataset from three hundred to three thousand images, significantly increasing the quantity and variability of the training sample while maintaining the fidelity of label information.

Augmentation was applied only to the training split and was designed to enhance discontinuity-level variability rather than to introduce new weld seams. This distinction aligns with the study’s objective of surface discontinuity detection, in which the weld bead primarily serves as a contextual class to support accurate localization. The augmented dataset, therefore, preserves the same 300 unique weld seams while diversifying the visual appearance of discontinuities across them.

### 2.9 Dataset analysis

**FIGURE 3:**
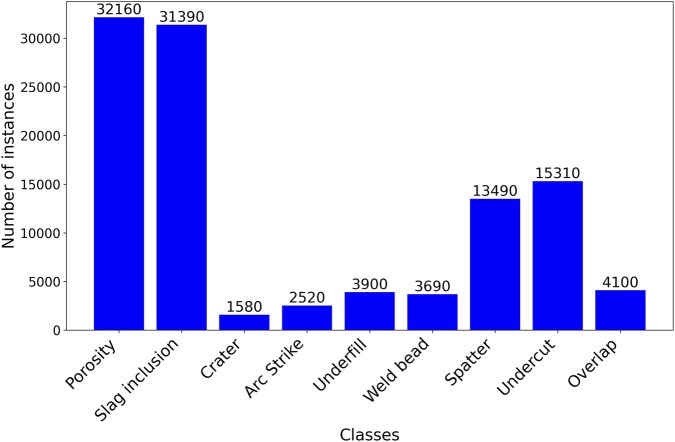
Distribution of annotated instances across classes in the SMAW dataset.

Following the data augmentation, 108,140 labeled instances were obtained across nine classes: eight different discontinuity types and the weld bead. The distribution of instances per class is shown in Figure 3, highlighting a higher occurrence of porosity and slag inclusions, followed by undercut, spatter, and weld bead. Less frequently annotated classes, such as crater, arc strike, and underfill, represent more subtle or less frequent discontinuities.

**FIGURE 4:**
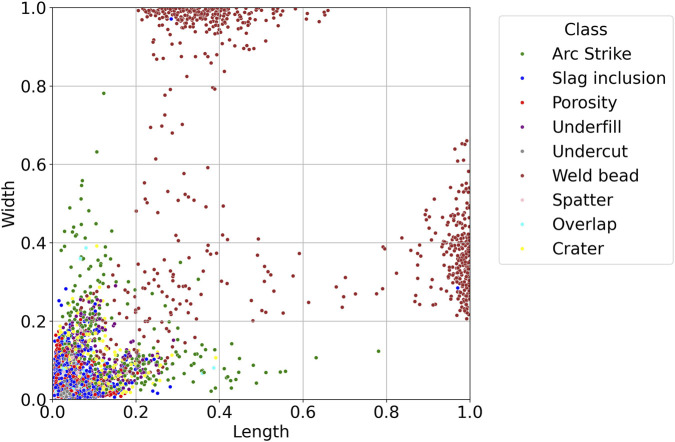
Normalized bounding box size for each class.

The labels' spatial characteristics were also examined. Figure 4 shows the relative bounding box dimensions normalized with respect to image size. Weld beads tend to occupy a larger spatial footprint, whereas discontinuities such as porosity, slag inclusions, and craters are typically smaller and more compact. This variation has implications for the model’s detection sensitivity across classes with different aspect ratios and scales.

**FIGURE 5:**
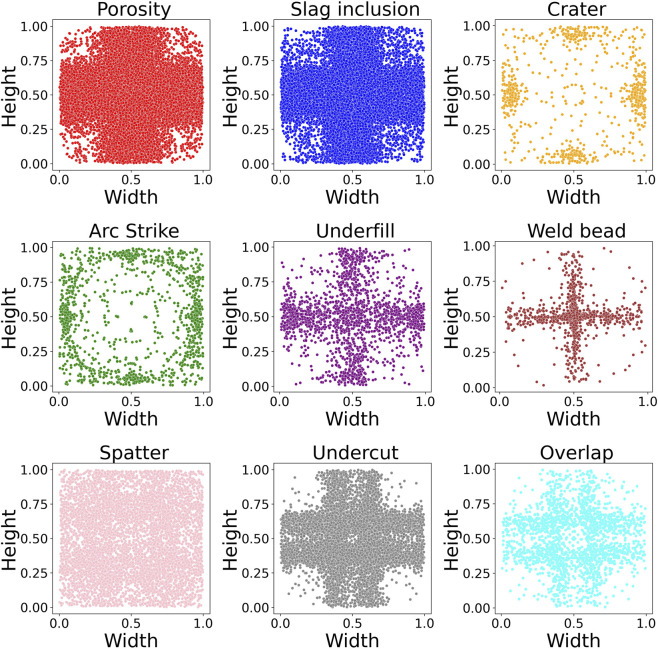
Spatial distribution heatmaps for all annotated classes.

Furthermore, Figure 5 shows the positional distribution of each class. Most classes are uniformly distributed across the weld surface, while others, such as weld bead and underfill, tend to align horizontally and vertically, reflecting their natural occurrence patterns during deposition. This visualization reinforces the variability and representativeness of the dataset for training robust object detection models.

### 2.10 Model selection and training configuration

For the surface discontinuities detection task, a YOLOv7-p5 architecture initialized from the official pretrained weights was employed. This initialization, based on the MS COCO dataset (Lin et al., 2014), was used to accelerate convergence and stabilize early-stage learning on the relatively small, specialized SMAW dataset. All layers were retrained on the SMAW data with no frozen parameters of COCO-specific label mapping, allowing the network to fully adapt to weld-specific textures and discontinuity patterns while benefiting from the general feature priors of the pretrained backbone.

Although pretraining can introduce domain bias when transferring from natural images to metallic surfaces, this initialization produced faster, more stable convergence than random initialization, which led to unstable early training (Yosinski et al., 2014; Chen et al., 2021). Therefore, pretrained initialization was retained for all experiments as the most effective configuration for our dataset size and computational resources.

A series of training runs were conducted using the augmented dataset to compare the model performance in terms of classification accuracy, convergence stability, and computational performance. All experiments were executed on a Dell Precision workstation with an Intel Core i9-13900K processor, 64 GB of RAM, and an NVIDIA RTX A5000 GPU under a Windows Subsystem for Linux (WSL) environment with open-source Python libraries.

Training parameters followed the recommended YOLOv7 configuration (Wang C. Y. et al., 2022), with minor adjustments to account for dataset size and class distribution. The main hyperparameters, including learning rate schedules, momentum, weight decay, and warm-up settings, are summarized in Table 3. Default values were confirmed to provide stable convergence in short validation trials, and no exhaustive grid search was performed due to the dataset size and available computational resources. Each training session was monitored throughout its epochs to evaluate model behavior, including loss evolution, prediction accuracy, and generalization capability. These evaluations were performed by tracking detection metrics such as precision, recall, and mean average precision, discussed in Section 2.11.

**TABLE 3:** Hyperparameters used to train the YOLOv7 models.

Hyperparameter	Value
Momentum	0.937
Initial learning rate (Lro)	0.01
Final learning rate (Lrf)	0.1
Weight_decay	0.0005
Warmup_epochs	3
Warmup_momentum	0.8
Warmup_bias_lr	0.1
Box	0.05

#### 2.10.1 Dataset partitioning

To train the YOLOv7 model, the augmented dataset of 3000 images was split into training, validation, and test subsets. Each subset comprised 70%, 20%, and 10% of the images, respectively. The division was performed using grouped, stratified sampling at the source-image level to prevent augmentation leakage. All augmented variants derived from the same source image were assigned to a single subset.

The partition procedure used a fixed random seed of 40, yielding 209, 61, 30 weld bead groups for training, validation, and testing, respectively. Distribution checks confirmed no leakage and slight drift between the test split and the overall dataset ( ≤ 4.59 pp in class proportions; ≤ 5.00 pp in electrode-type proportions).

The training and validation subsets were used exclusively for model optimization, threshold calibration, and convergence monitoring, while the test subset remained unseen until final evaluation (Section 3.3). All reported models were initialized from the official YOLOv7 pre-trained weights and retrained end-to-end on the SMAW dataset, following the configuration described in Section 2.10.

### 2.11 Evaluation metrics and monitoring

Model performance was rated using widely accepted object detection metrics. These metrics are derived from four fundamental classification outcomes: true positives (TP) that represent correctly identified instances of a discontinuity, false positives (FP) that represent non-discontinuity regions incorrectly identified as discontinuities, false negatives (FN) that are actual discontinuities that the model failed to detect, and true negatives (TN) that are correctly identified non-discontinuity regions. From these outcomes, the following core evaluation metrics were computed: precision (P), recall (R), and mean average precision (mAP) (Padilla et al., 2021). Precision is a metric used in object detection tasks to evaluate the accuracy of the model’s positive predictions. It helps determine the model’s reliability in identifying positive instances by minimizing false positives. Higher precision indicates a lower rate of falsely predicted positive instances, as shown in Equation 1.

$$P=\frac{TP}{TP+FP}$$ (1)

Recall measures the proportion of actual positive instances correctly identified by the model. Recall quantifies the model’s ability to detect and capture object instances correctly. A higher recall indicates a lower rate of missed detections, as shown in Equation 2.

$$R=\frac{TP}{FN+TP}$$ (2)

The mean average precision (mAP) is the primary metric for evaluating object detection models. It summarizes the precision-recall curve across different confidence thresholds. In this study, two versions were monitored: mAP@0.5, computed at an Intersection over Union (IoU) threshold of 0.5, and mAP@0.5:0.95, averaged over IoU thresholds ranging from 0.5 to 0.95 in steps of 0.05. As shown in Equation 3, mAP can be approximated by averaging the Average Precision (AP) values across all object classes.

$$mAP=\frac{1}{N}\sum_{q=1}^{N}AP\left(q\right)$$ (3)

In this context, AP(q) is the average precision for the q-th class, and N is the total number of classes evaluated. This metric summarizes the model’s effectiveness in detecting multiple classes simultaneously, accounting for precision and recall across varying confidence thresholds.

The evolution of these metrics was tracked throughout training using the built-in monitoring tools of the YOLOv7 framework. Figures 7, 8 shows examples of the learning curves of different YOLOv7 models for the training and validation sets, including losses (box, objectness, and classification), recall, mAP@0.5, and mAP@0.5; 0.95. These metrics were used to evaluate convergence, detect potential overfitting, and guide model selection based on validation performance.

#### 2.11.1 Threshold calibration and test-time protocol

In this study, we set the Non Maximum Suppression (NMS) IoU to 0.45, following the standard YOLOv7 implementation defaults and prior YOLO-style practice (Gählert et al., 2025; Wang C. Y. et al., 2022; Liu et al., 2023), with only the confidence threshold being tuned on the validation subset. The confidence threshold was swept from 0.05 to 0.5 in increments of 0.5, and the operating point was selected by maximizing macro F1 on the validation subset. This procedure yielded confidence = 0.20. The selected thresholds were then fixed and applied once to the test subset for final reporting. Alongside threshold-dependent metrics like precision and recall, it is reported threshold-independent scores (mAP@0.5, mAP@0.5:0.95), per-class AP/precision/recall, and a test subset confusion matrix are reported to characterize class-specific behavior and error modes.

## 3 Results

This section presents the training and evaluation results of YOLOv7 models for surface discontinuities detection in the SMAW process. Firstly, the impact of dataset size and epoch count on detection accuracy is evaluated to determine optimal training parameters. Then, the model’s predictive capability is examined through dataset image evaluations. Finally, the model’s generalization ability is explored using external images from publicly available data.

### 3.1 Dataset size

To evaluate the impact of dataset size on the validation performance, the YOLOv7-p5 architecture was trained using three subsets of the original weld bead image database: 300, 1500, and 3000 images. The learning rate, batch size, and number of epochs were kept constant to isolate the effect of the training set size. All reported parameters in this subsection correspond to the validation subset evaluated during training and were used exclusively for model selection and parameter tuning. The results are summarized in Table 4, which shows the model identifier, number of epochs, dataset size, batch size, final precision, mAP@0.5, and total training time for each case.

**TABLE 4:** Effect of dataset size on precision, mAP, and training time for YOLOv7 models.

Model	Epochs	Dataset size	Batch size	Precision	Recall	mAP@0.5	mAP@0.5:0.95	Training time
A	300	300	16	32%	30%	26%	13%	0:38:42
B	300	1500	16	93%	81%	87%	51%	4:01:40
C	300	3000	16	97%	91%	94%	68%	6:17:24

A substantial improvement in precision and mAP was observed with increasing dataset size, as shown in Table 4. Precision rose from 32% to 93% when the training subset expanded from 300 to 1500 images, and mAP@0.5 increased from 25% to 87%. This sharp improvement indicates a representativeness threshold: with only three hundred images, several rare discontinuities (arc strike, underfill, and crater) were underrepresented, as only a few instances of these classes were recorded in the original dataset. Even in the expanded dataset, their frequencies remain lower than those of more common classes such as porosity or spatter, which helps explain the residual performance gap at larger sample sizes. Expanding to 1500 images increased the number and diversity of label instances, improving class balance and reducing false positives, as shown in Figure 3. Further enlargement of 3000 images yielded smaller but consistent gains, with precision = 97% and mAP@0.5 = 94%, confirming that performance depends strongly on sample diversity and augmented discontinuity coverage.

The expansion to 3000 images was achieved through controlled data augmentation applied only to the training subset, enriching discontinuity-level variability without introducing new weldments. Importantly, the objective of this work is to detect surface discontinuities, with the weld bead labeled primarily to provide spatial context and improve localization; the number of unique weld seams remained three hundred.

Additionally, a well-defined increase in training time was observed with a larger dataset. Training the model with 300 images required approximately 40 min, whereas training with 1500 images extended to about 4 h, and training with 3000 images took more than 6 h. This behavior shows the computational cost associated with dataset scaling, which must be considered when using environments with limited hardware resources or strict training time constraints.

### 3.2 Epoch count

After analyzing the general effect of dataset size, the influence of training duration on the validation subset performance was analyzed to identify whether extended training offered additional benefits. The model’s precision was evaluated at four key training checkpoints: 150, 200, 250, and 300 epochs.

All training runs employed a YOLOv7-p5 architecture initialized from the official pretrained weights, following standard YOLOv7 practice. No layers were frozen, and all weights were retrained on the SMAW dataset to ensure adaptation to weld-specific textures and discontinuities.

Pretrained initialization is widely recognized to improve convergence stability and sample efficiency in object-detection tasks (Yosinski et al., 2014; Morales et al., 2018). This configuration enabled stable convergence within 200–300 epochs without overfitting, supporting its suitability for the relatively small SMAW dataset.

The results summarized in Table 5 correspond to validation metrics computed during training. These values were used exclusively for selecting the optimal number of epochs before testing on the independent subset presented later in Section 3.4.1.

The most substantial gains in precision and mAP, occur within the first 200 epochs. Training for 150 already yielded a precision of 94% and mAP, of 88%. Extending training to 200 epochs improves precision to 95% and mAP, to 92%, while training for 250 to 300 epochs led to only marginal additional gains, with precision stabilizing at 97% and mAP, increasing slightly from 93% to 94%.

**TABLE 5:** Validation subset precision achieved by YOLOv7-p5 at different epoch counts.

Model	Epochs	Precision	mAP@0.5	Training time
D	150	94%	88%	2:54:32
E	200	95%	92%	3:54:21
F	250	97%	93%	4:51:18
C	300	97%	94%	6:17:24

**FIGURE 6:**
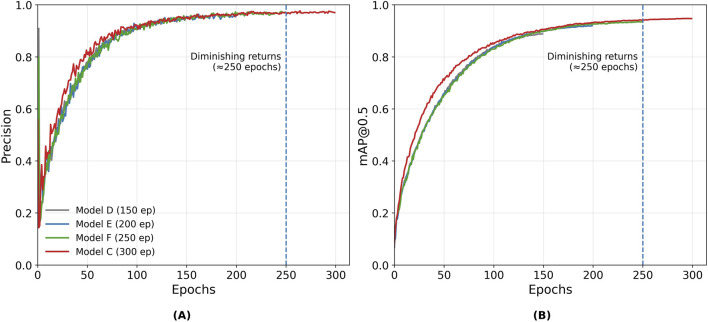
Evolution curves for YOLOv7 models. **(A)** Precision curve **(B)** mAP@0.5 curve. Validation metrics were computed using EMA weights maintained during training, and the dashed line at 250 epochs highlights the onset of diminishing returns in validation performance.

The precision and mAP evolution curves shown in Figure 6 were obtained from the validation subset at regular checkpoints using the exponential moving average (EMA) weights maintained during YOLOv7 training (Wang C. Y. et al., 2022). The EMA mechanism smooths short-term oscillations in the model parameters, providing a clearer view of convergence behavior and supporting a consistent assessment of stability and diminishing returns across epochs (Morales-Brotons et al., 2024). The curves show that performance improvements plateau after approximately 250 epochs, marking the onset of diminishing returns, where further training increases computation cost without yielding meaningful accuracy gains.

**FIGURE 7:**
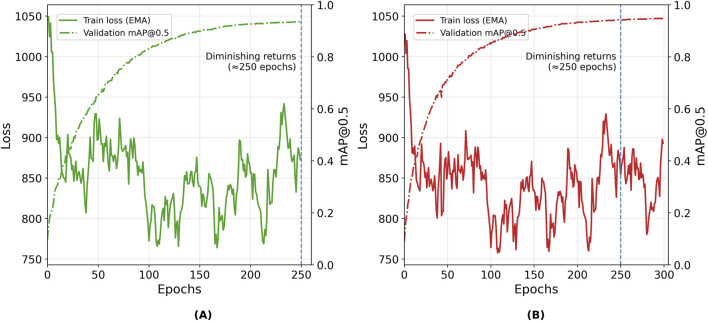
Training box-loss (solid) and validation mAP@0.5 (dashed) versus epochs for YOLOv7-p5 models: **(A)** Model F (250 epochs) and **(B)** Model C (300 epochs). The dashed line at 250 epoch indicates the saturation point in learning.

To further examine convergence behavior, the complete training and validation histories of Model F (250 epochs) and Model C (300 epochs) are presented in Figure 7. These two configurations were selected as representative cases: Model F corresponds to the epoch count where validation metrics have already stabilized, while Model C extends training beyond this point to confirm that the model maintains stable performance. Both models exhibit smooth and consistent trends across training and validation metrics, indicating that the model converge reliably without divergence between the loss and mAP@0.5 curves.

**FIGURE 8:**
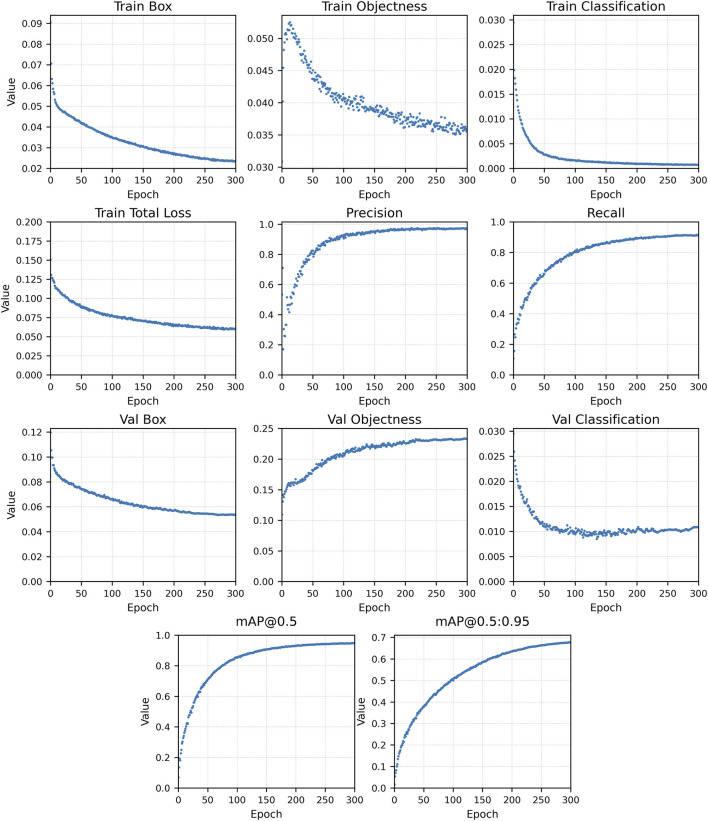
Complete training and validation evolution curves of Model C (YOLOv7-p5, 300 epochs, 3000-image dataset).

To provide additional context, Figure 8 shows the detailed evolution of all monitored metrics for Model C across the full 300 epoch training period, including loss components, precision, recall, and mAP metrics. Together, these results confirm that extended training beyond 250 epochs primarily refines weights without improving generalization.

As shown in Table 5, Model F (250 epochs) and Model C (300 epochs) achieved the highest validation performance, both exhibiting stable convergence and no overfitting. Model F reached precision = 97% and mAP@0.5 = 93% with a total training time of 04:51:00, whereas Model C required 06:17:00 to complete 300 epochs and improved mAP@0.5 by only one percentage point. These results confirm that extending training beyond 250 epochs yields limited accuracy gains relative to the increase in computational cost.

Nevertheless, Model C was selected as the final configuration for independent test subset evaluation (Section 3.3) because it provided the most consistent validation metrics and complete convergence across all monitored losses.

### 3.3 Model prediction evaluation

After selecting Model C based on validation performance, its generalization capability was evaluated on the independent test subset. All results in this section correspond exclusively to unseen data and quantify the model’s predictive performance under the fixed operating point determined during validation, with conf = 0.20 and NMS IoU = 0.45 (see Section 2.11.1).

#### 3.3.1 Operating point and test subset performance

Using the fixed operation point, Model C achieves mAP@0.5 = 24% and mAP@0.5:0.95 = 13% on the test subset, with precision = 45%, recall = 31% and macro F1 = 37%, as shown in Table 6. The performance exhibits a precision-oriented behavior, demonstrating effective false-positive control but limited recall due to small, low-contrast surface discontinuities typical of SMAW welds.

**TABLE 6:** Operating point and test subset performance.

Subset	Conf	NMS IoU	Precision	Recall	F1	mAP@0.5	mAP@0.5:0.95
Test	0.20	0.45	45%	31%	37%	24%	13%

The reduction in mAP from validation (94%) to test (24%) reflects the expected domain gap between the training/validation weld seam and previously unseen weldments. This behavior confirms that while the model captured representative discontinuity features, generalization remains constrained by the number and diversity of independent samples.

#### 3.3.2 Per-class test metrics

Table 7 lists the class-wise AP@0.5 scores. The highest performance was achieved for the weld bead (85%) and spatter (54%), followed by moderate values for arc strike, overlap, and crater. Lower values for porosity, underfill, undercut, and slag inclusion reveal that detection reliability scales with object size and contrast: significant, well-defined discontinuities are detected more consistently, whereas minor or edge-like defects remain recall-limited.

**TABLE 7:** Per-class test metrics at conf = 0.20, IoU = 0.45.

Class	AP@0.5
Weld bead	85%
Spatter	54%
Arc Strike	34%
Crater	13%
Overlap	20%
Underfill	7%
Porosity	4%
Undercut	3%
Slag Inclusion	0.2%

#### 3.3.3 Confusion matrix

**FIGURE 9:**
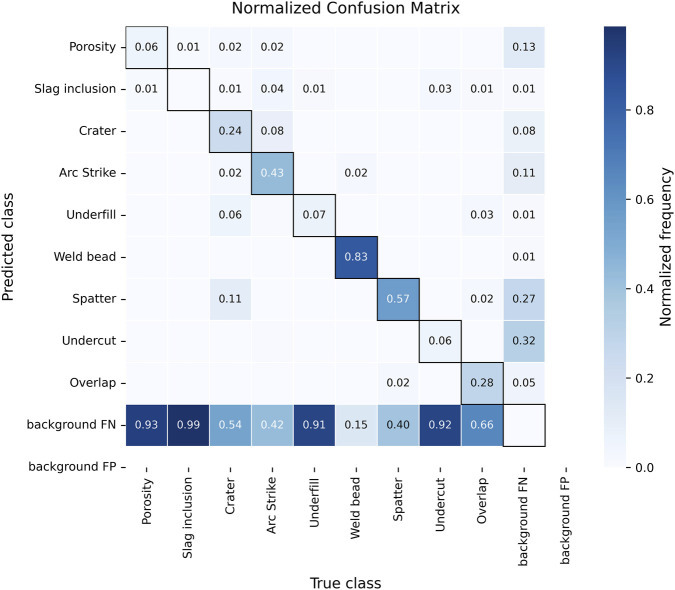
Confusion matrix on the test subset at conf = 0.20, NMS IoU = 0.45.

The confusion matrix presented in Figure 9 shows that the test subset background false positives and false negatives dominate the overall error distribution rather than cross-class confusions. False positives are concentrated in spatter and undercut, often triggered by reflective textures or weld toe irregularities. False negatives occur mostly in porosity, slag inclusion, underfill, and undercut, which have a small size and low visual contrast. Additionally, cross-class confusions appear sporadically between crater-arc strike and undercut-overlap, where similar geometry and adjacency effect can cause NMS suppression of valid detections.

The test subset results indicates that class separation is robust; however, recall for small-scale discontinuities remains the primary limitation. Future work should explore other methodologies to improve small-defect sensitivity.

### 3.4 Validation and robustness evaluation

The following subsection presents a qualitative and robustness evaluation of Model C. Unlike the previous section, which reported quantitative results on the independent test subset, these analyses utilize validation images to visualize prediction behavior, assess sensitivity to image geometry, and evaluate generalization to external datasets.

#### 3.4.1 Dataset image evaluation

The performance of Model C was qualitatively evaluated on the validation subset of the augmented dataset, to visually assess the localization and classification of surface discontinuities. To ensure the model’s robustness across different welding skill levels and conditions, the dataset included welds produced by operators of varying expertise: engineering students, general welders, and certified welders. This approach introduced realistic variability in bead quality, surface texture, and discontinuity occurrence.

**FIGURE 10:**
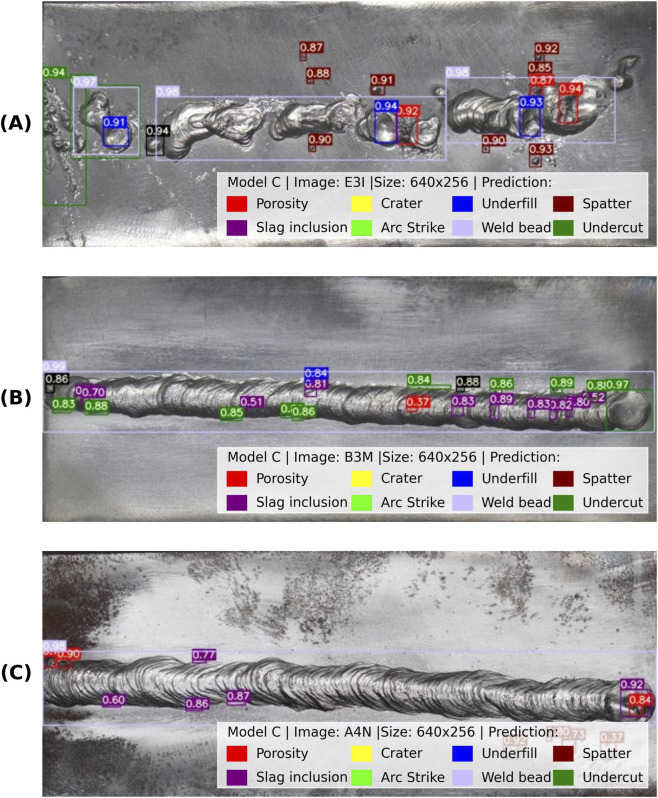
Detection results on validation dataset images produced by welder’s experience: **(A)** engineering student **(B)** general welder **(C)** certified welder.

Figure 10 presents representative results for each operator category. Model C correctly identified multiple discontinuity types across all examples, including porosity, slag inclusion, arc strike, spatter, underfill and weld bead geometry. The bounding boxes were closely aligned with the ground truth locations, and confidence scores were high even in more challenging cases, such as irregular weld patterns typical of less experienced welders. Figure 10A shows prediction for a weld bead produced by an engineering student, characterized by irregular geometry and abundant surface discontinuities. Figure 10B shows a weld bead produced by a general welder, exhibiting moderate discontinuity occurrence. Figure 10C shows a weld bead produced by a certified welder, where the model correctly detected the nearly discontinuity-free surface region. Notably, in Figure 10C, the model shows its ability to detect discontinuities and recognize discontinuity-free regions accurately. This dual capability is essential for practical automated inspection systems, ensuring that discontinuity detection and weld quality verification are reliable.

While the model shows good performance, challenges were noted in detecting overlapping discontinuities like porosity near slag inclusion and small discontinuities on uneven surfaces. These limitations suggest opportunities for future improvements through further dataset expansion or fine-tuning.

#### 3.4.2 Aspect ratio evaluation

**FIGURE 11:**
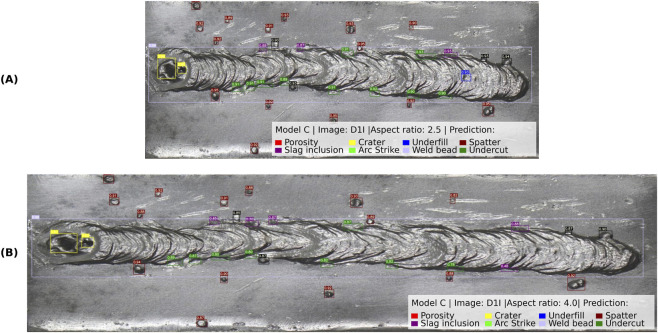
Aspect ratio evaluation of dataset images. **(A)** Model prediction for a 2.5:1 aspect ratio image; **(B)** prediction for a 4.0:1 aspect ratio image.

To assess the robustness of Model C to geometric variations in image acquisition, an additional evaluation was conducted using validation images with modified aspect ratios. Aspect ratio variations can introduce challenges in the model as the images can be captured using different camera models or configurations. For this evaluation, a representative weld bead image from the validation set was resized to a 2.5:1 aspect ratio as shown in Figure 11A and a 4.0:1 aspect ratio as shown in Figure 11B.

The model detected multiple discontinuities in both cases, including porosity, slag inclusion, underfill, crater, arc strike, and weld bead geometry. The bounding boxes remained consistent, and the confidence score stayed within acceptable margins. However, a slight reduction in confidence was noted in the 4.0:1 aspect ratio image, particularly for small discontinuities such as underfill and slag inclusion, dropping to 0.66 in isolated cases.

These results show that the model is relatively robust to moderate aspect ratio variations, maintaining spatial consistency and class confidence within expected tolerances. However, as the aspect ratio increases further, minor degradations in detection confidence and bounding box precision become evident. These findings suggest that training with augmented aspect ratios or multi-scale normalization could further enhance the model’s adaptability to varying image geometries.

#### 3.4.3 External image evaluation

An evaluation using external images that were not part of the dataset was conducted to further assess the generalization ability of the selected Model C. This test aimed to simulate real-world deployment scenarios, where weld images may come from different equipment, settings, or environments.

The images were sourced from a publicly available dataset on Kaggle (Wijaya, 2024). Images under different lighting and surface conditions compared to the original dataset were used in this study. These differences introduce additional challenges, such as variability in weld bead appearance, noise, and discontinuities contrast.

Before testing the images, a pre-processing pipeline was implemented to ensure the external images were compatible with the input configuration. In this regard, only images that matched a similar plane shot were selected for testing. External images captured at angles, representing different welding processes, or involving different types of joints, were excluded to maintain consistency with the original dataset characteristics. Additionally, selected images were cropped to isolate the weld bead region and then resized to match the input dimensions of the original dataset.

**FIGURE 12:**
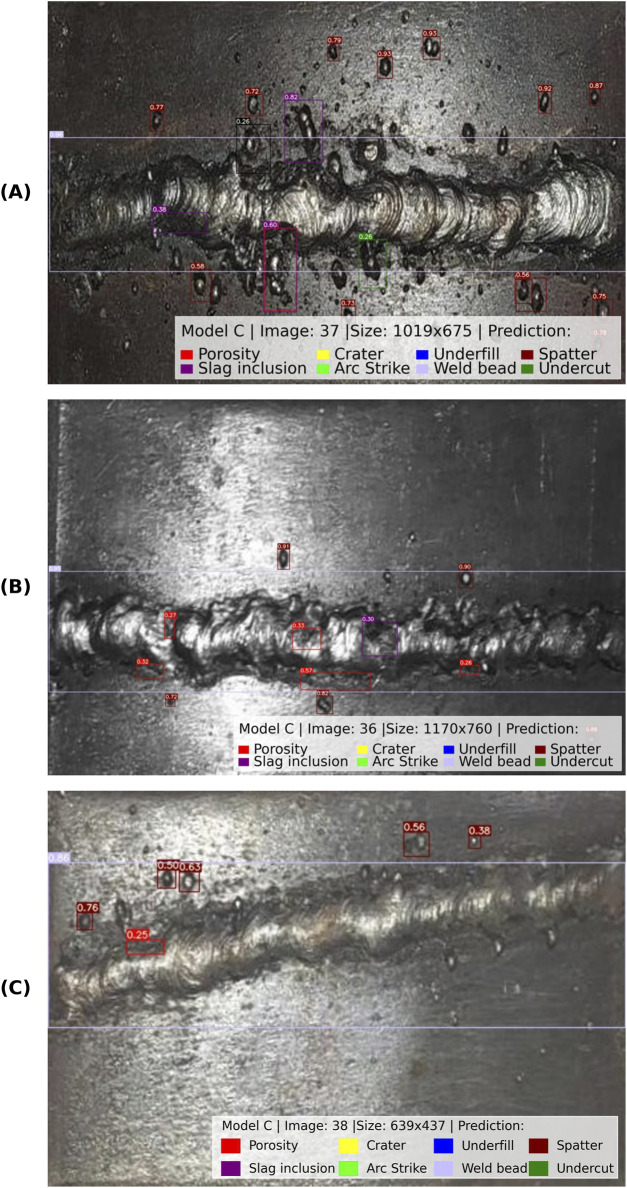
Detection results of model C for external images sourced from the Kaggle dataset (Wijaya, 2024). **(A)** Image sized 1019 x 675, **(B)** Image sized 1170 x 760, **(C)** Image sized 639 x 437.

Figure 12 shows the detection results for three representative external images from the Kaggle dataset. The selected images enable evaluation of the model’s performance when predicting images of different sizes, lighting conditions, surface texture, and weld bead orientation. When evaluated on these images, Model C achieved an overall precision of 65.2%, a recall of 25.2%, and an mAP@0.5 of 26.3% as it is shown in Table 8. Among the annotated classes, the weld bead was detected with high accuracy, while porosity and overlap showed moderate reliability. In contrast, spatter and undercut detection were inconsistent, and no predictions were reported for slag inclusion, crater, arc strike, or underfill, as these discontinuities were not annotated in the selected images according to the dataset’s labeling protocol.

**TABLE 8:** External dataset evaluation results (only the images presented in Figure 11).

Class	Precision (%)	Recall (%)	mAP@0.5 (%)
All	65.2	25.2	26.3
Porosity	0.0	0.0	0.3
Weld bead	83.7	100.0	99.6
Spatter	42.6	13.5	16.5
Undercut	100	0.0	0.0
Overlap	99.9	12.5	15.0

Undercut class report precision = 100% with recall and mAP, 0%.

This outcome results from class imbalance and the limited test set size: only one undercut instance was annotated across the three Kaggle images used for external testing. The model produces very few predictions without false positives (yielding high precision), but none overlapped with the annotated ground truth under the IoU ≥ 0.5 threshold, resulting in zero recall and mAP.

It is essential to note that, since the external images were cropped and resized, and the physically welded plates were not available for validation, the ground truth labels relied on public annotations, which may contain inconsistencies. This limitation, combined with the restricted number of evaluated images, introduces uncertainty in the reported quantitative metrics. Nevertheless, the observed results confirm the model’s ability to generalize to weld with unfamiliar textures and illumination, albeit with reduced recall. These results highlight the need for expanded cross-dataset evaluation and domain adaptation strategies to ensure robust deployment across diverse acquisition settings.

## 4 Discussion

The results of this study demonstrate that the YOLOv7-p5 model can effectively identify multiple surface discontinuities produced during SMAW welding. Model performance was first assessed on the validation subset to determine the influence of dataset size, epoch count, and other optimization factors. The final configuration (Model C) was subsequently evaluated on the independent test subset (Section 3.3) to verify generalization to unseen weldments and distinct acquisition conditions. The observed variability in bead texture and illumination also reflects the inherent complexity of SMAW welds (e.g., flux-generated slag residues and manual operator variability), making SMAW surface conditions distinct from gas-shielded processes (GMAW/GTAW) and supporting the need for process-specific datasets.

### 4.1 Dataset size and training duration

Increasing the dataset size and the number of training epochs significantly improved detection accuracy. Expanding the training subset from 300 to one 1500 increased precision from 34% to 93%, confirming that dataset representativeness strongly affects model generalization, particularly for rare discontinuity classes such as arc strike, underfill, and crater (Table 5). Beyond 250 epochs, however, performance gains became marginal: both training and validation losses plateaued while validation mAP@0.5 remained stable (Figures 6–8), confirming convergence without overfitting. These findings are consistent with prior reports (Yang L. et al., 2021; Liu and Wang, 2023) that larger and more diverse datasets improve model generalization. Based on the observed convergence behavior, training was limited to 300 epochs to balance accuracy and computational cost.

Future dataset expansion will continue within the SMAW process, incorporating fillet and butt-joint geometries to capture additional discontinuity modes beyond the bead-on-plate configuration used in this study.

### 4.2 Aspect ratio and external-dataset behavior

The aspect ratio evaluation (Section 3.4.2) showed that the model is robust to moderate geometric changes but highlighted reduced confidence at higher ratios, an important consideration for variable field camera setups. Other studies (Wang C. et al., 2022; Yang et al., 2023) have similarly noted the importance of image preprocessing and input consistency for optimal model performance. In the external dataset evaluation (Section 3.4.3; Table 8; Figure 12), the model retains its detection capability, but shows lower recall and a drop in confidence scores due to domain shift and annotation inconsistencies. Despite these limitations, the correct localization of major discontinuities was maintained, demonstrating satisfactory robustness.

### 4.3 Training strategy and domain adaptation

The model was initialized from the official YOLOv7 pre-trained weights, and all layers were retrained on SMAW-specific images. This configuration enables faster convergence and stable optimization, while allowing for complete adaptation to weld textures and surface morphology. Previous studies have demonstrated that initializing networks with transferred features can enhance generalization and reduce training time, even when the source and target data domains differ (Yosinski et al., 2014). In this study, full network retraining ensured that both low- and high-level features adapted to the SMAW domain. Future work will explore transfer learning and domain adaptation strategies based on welding-oriented pre-trained models to improve cross-process generalization further.

### 4.4 Error sources and class imbalance

Despite robust in-dataset performance, some limitations remain. The model occasionally produced lower confidence scores or partial bounding boxes in dense weld regions with overlapping discontinuities or occlusion. These challenges are common in object detection tasks and are exacerbated by class imbalance, as discontinuities such as spatter and porosity occur more frequently than other types, as shown in Figure 3. While data augmentation partially mitigated this issue, the results indicate that further expansion with multi-source and field-acquired images is needed to improve balance and strengthen generalization. Another limitation concerns the absence of formal inter-annotator agreement metrics; although labels were created by consensus, a quantitative IAA assessment is planned for future dataset revision to strengthen reproducibility.

### 4.5 Comparative analysis with related works

To contextualize the performance, Table 9 compares the proposed model, YOLOv7-p5, with recent weld-defect detectors across different welding processes and dataset sizes. Reported metrics reflect the evaluation split used in each study: YOLO-MSAPF (Wang et al., 2023) reports on its validation subset of GMAW images; WeldNet (Wang et al., 2024) reports on a held-out test subset of 33254 TIG images; (Xu and Li, 2024); report averages over internal test subsets from multiple splits (6:3:1–8:1:1); and this work reports validation results for model selection and provides an independent test-subset evaluation. Unlike prior works centered on GMAW/TIG, our study contributes an annotated SMAW dataset (3000 images, nine classes). It shows that process-specific data and training optimization can match or exceed the accuracy of an architecturally enhanced model while maintaining practical efficiency.

**TABLE 9:** Comparison of the proposed YOLOv7-p5 model with state-of-the-art weld-defect detection approaches.

Study	Model	Welding process	Dataset size	No. of classes	Precision (%)	mAP@0.5 (%)
Wang et al. (2023)	YOLO-MSAPF	GMAW	7580	8	≈ 95	95
Wang et al. (2024)	WeldNet (+FE + KD)	TIG	33254	6	83.8	(N/A)
Xu and Li (2024)	Improved YOLOv7 (+Le-HorBlock + CoordAtt + SIoU	GMAW	2000	3	96.5	78.6
This work (2025)	YOLOv7-p5	SMAW	3000	9	97	94

Metrics are reported on validation or internal-test splits as in the original papers.

### 4.6 Interpretation of external class anomalies

In the external dataset evaluation, the Undercut class reported contradictory results, such as precision values of 100% with recall and mAP equal to 0%. This effect arises from the minimal size of the test set (three images) and the sparse distribution of annotated discontinuities. Precision is determined by the absence of false positives among the model’s predictions, whereas recall and mAP depend on the overlap between predictions and ground-truth annotations. In this case, the model produced few predictions that were not contradicted by false detections, yielding perfect precision, but no matches under the IoU threshold with the annotated discontinuities, resulting in zero recall and mAP. These values should therefore be interpreted cautiously due to the dataset limitations, rather than as a true reflection of model performance for these classes.

### 4.7 Practical deployment and future directions

While the present work focused on offline image-based detection to support visual inspection tasks, real-time inference was not evaluated. The intended application at this stage is a complementary tool. Once an image is captured, the model predicts the presence and location of discontinuities, and then the results are validated by a certified welding inspector. Nevertheless, enabling real-time operation remains an important future direction, particularly when coupled with a defect decision framework. Real-time operation would allow the system to be deployed on edge devices, enabling inspection in areas with limited accessibility and reducing dependence on post-processing. Such integration could enhance field usability, providing inspectors with immediate feedback and facilitating continuous monitoring in production environments.

Building on this perspective, it is important to consider how the model’s predictions relate to human inspectors. The present evaluation relied on a labeled dataset as the reference standard; no direct quantitative comparison with human inspectors was conducted. Such a comparison would help assess the model’s practical utility in real inspection scenarios. Rather than comparing the number of individual discontinuities detected, which would effectively reduce the evaluation to a bounding-box count, a more representative approach would be at the joint level. In this framework, both the model and inspectors would provide a pass/fail decision for the weld segment based on detected discontinuities and applicable acceptance criteria. This strategy aligns with the planned integration of a defect decision framework and would enable a direct comparison with the holistic inspection process used by certified welding inspectors.

From a methodological standpoint, future improvements could also be achieved by integrating segmentation-based architectures such as Mask R-CNN (He et al., 2017) and DeepLab (Chen et al., 2016), which could further enhance this applicability. Unlike detection-only models, segmentation methods provide pixel-level localization of discontinuities, enabling more accurate quantification of defect size, shape, and orientation. This capability is particularly relevant when assessing discontinuities in the context of code compliance, where defect classification often depends on measured size and extent. Such integration is especially significant to AWS D1.1 Structural Welding Code (Table 8) (American Welding Society, 2020a), which established visual inspection acceptance criteria. Under this framework, the classification of a discontinuity as a defect is not based solely on its presence but also on whether its dimensions exceed defined thresholds. Combining detection with segmentation allows the system to evolve from a discontinuity identification tool into a decision-support framework capable of guiding automated pass/fail evaluations in inspection workflows.

Finally, it is important to clarify the detection scope of the proposed model. In this work, a convolutional neural network model was trained to detect surface irregularities in SMAW weld seams, which are generally considered discontinuities. According to AWS A3.0 (American Welding Society, 2020b), discontinuity is an interruption of the typical structure of a material, such as a lack of homogeneity in its mechanical, metallurgical, or physical characteristics, and it is not necessarily a defect. In contrast, a defect is defined as a discontinuity or set of discontinuities that, by nature or accumulated effect, render a part or product unable to meet applicable standards or specifications. Within this framework, the model visually detects eight types of discontinuities as described in Section 2.7. However, some discontinuities (slag inclusions, lack of fusion, craters, and arc strikes) may be considered defects depending on specific code requirements. As a result, the proposed model should be regarded as a discontinuity detection tool, providing inspectors with reliable information that must be evaluated against acceptance criteria to determine whether a defect is present. This distinction highlights the model’s role as a complementary instrument for more complete and consistent visual inspection.

## 5 Conclusion

This study successfully developed and validated a YOLOv7-based deep learning model tailored for detecting surface discontinuities in the SMAW process. The following key conclusions were drawn:

1. The dataset size was crucial to the model’s performance. Model A, trained on 300 images, achieved limited precision (32%), recall (30%), and mAP@0.5 (26%), highlighting the dataset’s under-representation and lack of discontinuity variability. In contrast, Models B and C, trained with 1500 images and 300 images, respectively, achieved substantially higher performance, with Model C reaching 97% precision, 91% recall, mAP@0.5 of 94%, and mAP@0.5:0.95 of 68%. These outcomes confirm the strong dependence of detection performance on dataset size and diversity, emphasizing the need for representative SMAW imagery covering all discontinuity types.
2. An optimal training range was identified between 200 and 300 epochs, balancing detection performance with computational efficiency. As shown in Figures 6–8, training and validation losses stabilize beyond 250 epochs, while the validation mAP@0.5 plateaus, indicating convergence without overfitting and diminishing returns.
3. The model achieved robust in-dataset performance across welds produced by operators of different expertise levels, reflecting good consistency. However, an external evaluation of the Kaggle dataset showed reduced precision and recall, partially due to domain shift, illumination differences, and annotation inconsistencies, as discussed in Section 3.4.3.
4. While the model exhibited good overall generalization ability, detection of overlapping or occluded discontinuities in noisy weld regions remained challenging, particularly for spatter and porosity. This limitation, primarily related to class imbalance, underscores the need for dataset expansion with multi-source and field-acquired images to enhance generalization.
5. The system was designed as a complementary offline inspection tool rather than a real-time edge deployment. Future developments will consider real-time inference and decision-support frameworks, enabling deployment in environments with limited access.
6. No direct quantitative comparison with human inspectors was performed. A more representative approach would be a joint-level evaluation where both inspectors and the model provide pass/fail decisions based on applicable acceptance criteria. This strategy aligns with the planned development of a decision framework for defects.
7. Comparison with advanced models (Wang et al., 2023; 2024; Xu and Li, 2024) shows that, although architectural improvements improve accuracy, dataset engineering and domain-specific tuning remain equally critical.

Future work will focus on:

- Expanding the dataset with multi-source and field-acquired images to improve class balance and generalization; future releases will also include fillet and butt-joint welds to capture additional discontinuity modes beyond bead-on-plate configurations.
- Applying transfer learning with welding oriented pretrained networks to enable generalization from SMAW to other arc-welding processes while retaining accuracy on SMAW surface discontinuities.
- Integrating the detection with segmentation-based architectures to enable precise discontinuity quantification and support automated pass/fail decision in accordance with visual inspection acceptance criteria defined in AWS D1.1.
- Conducting joint-level comparative studies with certified welding inspectors, evaluating pass/fail decisions based on detected discontinuities and acceptance criteria to assess the model’s practical utility in real inspection workflows; this work will serve as the foundation for a decision-support framework linking detection to code-based compliance.
- Exploring architectural enhancements (e.g., lightweight attention or multiscale fusion modules) to optimize detection accuracy and computational efficiency.

## Data Availability

The raw data supporting the conclusions of this article will be made available by the authors, without undue reservation.
